# Application of clinical PET imaging to human laryngeal squamous cell carcinoma xenografts

**DOI:** 10.3892/etm.2013.1200

**Published:** 2013-07-03

**Authors:** OU XU, XIAOMING LI, CHUNGUANG SHAN, XING YANG, LIN ZHANG, JINGMIAO WANG

**Affiliations:** 1 Department of Otorhinolaryngology, The Second Hospital of Hebei Medical University, Shijiazhuang, Hebei 050000, P.R. China; 2 Departments of Otorhinolaryngology Head and Neck Surgery, Bethune International Peace Hospital, Shijiazhuang, Hebei 050082, P.R. China; 3 PET Center, Bethune International Peace Hospital, Shijiazhuang, Hebei 050082, P.R. China

**Keywords:** laryngeal carcinoma, positron emission tomography, nude mice, xenograft, animal handling

## Abstract

Positron emission tomography (PET) imaging with [F-18]-fluoro-2-deoxy-D-glucose (^18^F-FDG) is extensively applied in clinical practice. However, in animal experiments, the application of clinical PET is difficult, due to limitations in sensitivity and spatial resolution. This study aimed to determine the potential of ^18^F-FDG PET with regard to the imaging of human laryngeal squamous cell carcinoma (LSCC) xenografts. Twenty-seven LSCC tumor-bearing nude mice were divided randomly into seven groups which were each handled differently; the anesthetization, fasting, warming and the time point at which scanning was initiated were varied. The size of each xenograft was measured prior to conducting the scan. Using the RAMLA 3D image reconstruction method, images were acquired. The region of interest (ROI) technology was adopted to calculate target and non-target (T/N) ratios. The results were subsequently analyzed by semiquantitative analysis. The analysis showed that there was no significant correlation between tumor size and PET image quality (r=0.381, P>0.05); however, the handling conditions of the mice had a greater influence on the tumor image quality. Fasting increased ^18^F-FDG uptake (T/N, 1.153±0.008) to a certain degree, although the effect was unstable. By contrast, combining warming and fasting increased ^18^F-FDG uptake significantly (T/N, 2.0±0.29; P<0.05). The acquisition time had no impact on the tumor image quality. The study demonstrated that the application of clinical PET scanning has potential in the study of human LSCC xenografts in nude mice, and that the quality of the image of the tumor is greatly influenced by the handling conditions of the animals.

## Introduction

Head and neck squamous cell carcinoma (HNSCC) is the sixth most common malignant tumor worldwide (1). Although therapeutic strategies have improved in in the past two to three decades, the overall five-year survival rate remains almost unchanged (2). The primary reasons for this are post-treatment locoregional recurrence and distant metastasis. The detection of tumor metabolism in the early phase is important when devising the individual therapeutic strategy and undertaking a prognostic evaluation. Traditionally, computed tomography (CT) and magnetic resonance imaging (MRI) have been used to clearly display anatomical structure. However, with regard to disease identification, evaluation of lymph node metastasis and prognosis, CT and MRI have certain limitations. Positron emission tomography (PET), a functional imaging technology, is extensively applied in clinical practice to detect tumors and evaluate cervical node metastases in patients with HNSCC, due to its high sensitivity and specificity and the fact that it enables the monitoring of the disease at a molecular level (3).

Although PET has important applications in clinical practice, the application of PET in animal experiments is difficult, due to limitations in sensitivity and spatial resolution. Consequently, micro-PET imaging has been designed for this purpose. Micro-PET overcomes the shortcomings of clinical PET and has been increasingly used in the imaging of murine models of human diseases. However, the application of micro-PET imaging is restricted, due to its expensive cost and single usage. The adaptation of clinical PET for use in animal studies is particularly challenging; resolution of this problem is likely to provide clinical PET with another valuable function, progress the clinical application of PET and reduce in the cost of scientific research.

To the best of our knowledge, the current study is the first to apply clinical PET to laryngeal squamous cell carcinoma (LSCC) xenografts. It is likely to provide a useful tool for exploring the mechanisms of tumor genesis and metabolism. In this study, we established an LSCC xenograft model in nude mice and utilized [F-18]-fluoro-2-deoxy-D-glucose (^18^F-FDG) as a tracer to study the quality of PET images under various conditions. By comparing the qualities of the images of the tumors, the most effective handling protocol was determined. The present LSCC xenograft study demonstrated further potential applications for clinical PET.

## Materials and methods

### Cell culture and animals

The present study was conducted at the Department of Otolaryngology Head and Neck Surgery in the Bethune International Peace Hospital (Shijiazhuang, China), and was approved by the Ethics Committee of the Bethune International Peace Hospital.

Hep-2 LSCC cells (Shanghai Life Science Academy, Chinese Academy of Science, Shanghai, China) were cultured in RPMI-1640 medium (Gibco BRL, Grand Island, NY, USA) which was supplemented with 10% fetal bovine serum (Hangzhou sijiqing biological engineering materials co., Ltd., Hangzhou, China), 1% glutamine and 0.5% HEPES. Cells were cultured at 37ºC in a humidified 5% CO_2_ incubator. The exponentially growing cells were harvested with 0.25% trypsin plus ethylenediaminetetraacetic acid, washed and suspended in phosphate-buffered saline (PBS). The number of cells was counted using a Coulter counter (Beckman Coulter, Inc., Brea, CA, USA).

All experiments were performed using 16–18 g male athymic NCr-nu/nu mice, purchased from the Academy of Military Medical Sciences Animal Center (Beijing, China). The nude mice were maintained and used according to guidelines of Hebei province laboratory animal management institutions and the experimental protocols were approved by the ethics committee of Bethune International Peace Hospital. Five animals were housed per cage and were maintained at a constant temperature and humidity in the Bethune International Peace Hospital Experimental Animal Center.

### Synthesis of ^18^F-FDG

^18^F-FDG was provided by the PET Center of the Bethune International Peace Hospital and was synthesized automatically by nucleophilic substitution on mannose triflate (1,3,4,6-tetra-O-acetyl-2-O-trifluoro-methanesulfonyl-β-D-mannopyranose) in a PET cyclotron unit.

### Self-made warming instrument

The warming instrument comprised a cabinet that was made of an extruding plate and sealed with adhesive tape. In this experiment, two cabinets were used to maintain the animals at a constant temperature. A larger cabinet was used for the animals that had been injected with ^18^F-FDG and were waiting for PET scanning, while a smaller cabinet was used for the animals that were undergoing PET scanning. The large and small cabinets measured 60×30×18 cm and 16×12×10 cm, respectively, with sight-holes measuring 24×16 cm and 6×4 cm, respectively. The bottom of the cabinet was paved with aluminum foil-reflecting film, with electrothermal film in the middle and a hole-type plastic bracket on the surface. A power line was welded to the electrothermal film and emerged from the side of the cabinet. At the opposite side, a temperature probe was inserted near the bracket, to enable the monitoring of the ambient temperature. A thermostat was situated outside the cabinet to regulate the temperature within.

### Establishment of the Hep-2 LSCC cell xenografts in the animals

Briefly, a 1×10^6^ cells/0.2 ml Hep-2-cell suspension was injected subcutaneously into unanesthetized mice. The experiments were typically performed 3–4 weeks later, when distributions of tumors ranging from 0.8 to 1.5 cm in diameter were observed on the backs of the mice. The average body weight of the mice was 23.9±1.3 g. Serum glucose levels were measured prior to the experiment in fasted mice, with the average value measured to be 4.7±0.5 mmol/l. Following this, the mice were selected randomly and divided into seven groups, each containing between three and six mice. There was no statistical difference in tumor size among these groups.

### Experimental procedures

The mice were studied under the experimental conditions summarized in Table I. Due to the small caliber of the murine tail veins, the administration of ^18^F-FDG by tail vein injection (intravenous) is challenging. Partial paravenous injection is common and has no significant influence on the biodistribution of ^18^F-FDG under different conditions. Therefore, ^18^F-FDG was injected intraperitoneally prior to PET scanning.

### Group A

The animals were fasted overnight, prior to measurements of weight and serum glucose being obtained. Following this, the animals were administered different doses of chloral hydrate at room temperature. There was one death immediately subsequent to the administration of 7 ml/kg 5% chloral hydrate. Two animals were anesthetized for 30–40 min following the administration of 5 ml/kg 5% chloral hydrate. When the dosage was supplemented with 2 ml/kg 5% chloral hydrate, the animals ceased breathing and died.

### Group B

Animals were fasted overnight and administered an isoflurane inhalation. Following the inhalation of isoflurane for ~10 sec, the animals rapidly lost consciousness; however, the effect was only maintained for 1 min. The animals were then injected with 2 ml/kg 5% chloral hydrate intraperitoneally, which resulted in anesthesia being maintained for 30–40 min. Following further isoflurane inhalation, the animals died.

### Group C

Animals had constant access to food and drinking water and were injected with 5 ml/kg 1% pentobarbital intraperitoneally. Following a short (~5 min) period of anesthesia, the animals lost consciousness and their breathing became deep and slow. The effect was maintained for 40–50 min. When the animals began breathing superficially, a further 2 ml/kg 1% pentobarbital was administered, which resulted in successful anesthesia. There were no animal deaths and therefore, in the following groups, the intraperitoneal injection of 1% pentobarbital was adopted as the primary anesthesia method. In group C, ^18^F-FDG [5–7 MBq (200 μCi) in 0.2 ml] was injected intraperitoneally once the animals were anesthesized with no warming and one hour later PET scanning was performed.

### Group D

Animals were fasted overnight, without any warming treatment, and were administered an intraperitoneal injection of ^18^F-FDG following a short period of anesthesia with pentobarbital. One hour later, PET scanning was performed.

### Group E

Animals were fasted overnight. Following a short period of anesthesia with pentobarbital, they were injected with ^18^F-FDG intraperitoneally and kept warm in the cabinet, where the temperature was 30ºC. One hour later, the mice were placed into the small cabinet and PET scanning was performed.

### Group F

The handling conditions of the animals were identical to those for group E. Following the intraperitoneal injection of ^18^F-FDG for 1.5 h, the animals were placed into the small cabinet and PET scanning was performed.

### Group G

The handling conditions of the animals were identical to those for group E. Following intraperitoneal injection of ^18^F-FDG for 2 h, the animals were placed into the small cabinet and PET scanning was performed.

### ^18^F-FDG PET imaging

The experiment was performed with the Philips ADAC Allegro™ PET scanner (Koninklijke Philips NV, Amsterdam, Netherlands). Subsequent to the animals being anesthetized and injected with ^18^F-FDG, they were fixed on the bracket with limbs stretched. According to the experimental group requirements, the animals were either warmed in the cabinet by thermostatic regulation or left without warming. The image data were collected by head position 3D mode and the acquisition time was 5 min per bed position. Through the RAMLA 3D image reconstruction method, coronal, sagittal and cross-sectional tomographical images were acquired. The region of interest (ROI) technology was adopted to calculate target and non-target (T/N) ratios, and the results were analyzed by semiquantitative analysis.

### Statistical analysis

The data are presented as the mean ± standard deviation (SD). Statistical comparisons were performed with SPSS 13.0 statistical software for Windows (SPSS, Inc., Chicago, IL, USA). The differences in the T/N ratios among the experimental groups were statistically evaluated by analysis of variance (ANOVA). P<0.05 was considered to indicate a statistically significant difference.

## Results

### Effect of anesthesia on the animals

In group A, different doses of 5% chloral hydrate were administered to the mice. Observation of the mice showed that a 7 ml/kg dosage (the highest limit for common usage) was lethal for the nude mice, while a 5 ml/kg dosage was only able to induce anesthesia for 30–40 min. If an additional dosage (2 ml/kg) was administered to the nude mice, the chloral hydrate was lethal. Isoflurane inhalation had a rapid effect on the mice (≥10 sec); however, the duration of the anesthesia was too short (≥1 min). When combining isoflurane inhalation with chloral hydrate injection, a synergistic effect was observed in the course of the anesthesia, although the combination was more dangerous. The administration of 1% pentobarbital (5 ml/kg) was relatively safe for the nude mice. The anesthesia had a relatively long duration (40–50 min), and it was possible to administer an additional dosage (2 ml/kg), according to the requirements of the experiment.

### Correlation between tumor size and PET image quality

In this study, the diameter of the tumors imaged by PET scanning ranged from 0.8 to 1.5 cm. A comparison between the results showed that there was no significant correlation between tumor size and PET image quality (r=0.381, P>0.05).

### Temperature change during anesthesia and PET

When animals were maintained under anesthesia for 30 min at room temperature (24ºC), the mean body temperature of the mice decreased from 31.72±0.46 to 24.2±0.12ºC. This marked reduction in body temperature was avoided when the mice were kept in the warming cabinet (body temperature subsequent to 30 min anesthesia, 33.01±0.77ºC). There was no mouse dehydration.

### Influence of different handling conditions on the ^18^F-FDG uptake of the tumor

**Figure 1:**
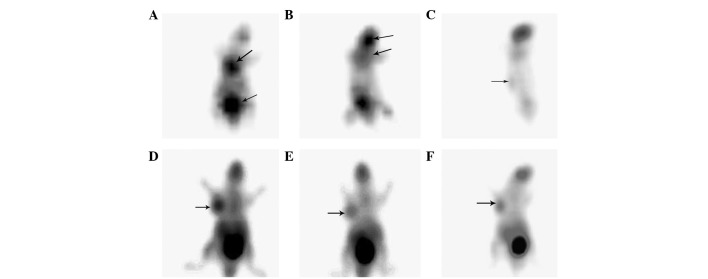
Positron emission tomography (PET) imaging of Hep-2 cell xenograft-bearing nude mice. (A) Group C (no fasting and no warming treatment): There was no imaging of the tumor; however, the images of the myocardium and urinary bladder were clear. (B) Group D (fasting and no warming): There was no imaging of the tumor; however, the images of the brain and brown fat were clear. (C) Group D (fasting and no warming): The image quality of the tumor was poor, with fuzzy edges and a low contrast; however, the image of the cerebral tissue was clear. (D) Group D [fasting and warming, 1 h subsequent to (F-18)-fluoro-2-deoxy-D-glucose (^18^F-FDG) injection]: The image of the tumor was clear and intact, and was highly contrasted with the adjacent tissue. (E) Group F (fasting and warming, 1.5 h subsequent to ^18^F-FDG injection): The image of the tumor was clear and the image quality was identical to that in group D. (F) Group G (fasting and warming, 2 h subsequent to ^18^F-FDG injection): The image of tumor was clear and there was no significant difference in image quality when compared with that of groups D and E.

Fig. 1 shows typical examples of the PET scans of the anesthetized animals acquired under the various conditions. With no warming and no fasting condition or fasting and no warming condition (Fig. 1A and B), the highest ^18^F-FDG uptakes were observed in the myocardium (T/N, 3.15±0.44), urinary bladder (T/N, 3.55±0.63), cerebral tissue (T/N, 3.1±0.60), skeletal muscle (T/N, 1.24±0.10) and brown fat (T/N, 2.55±0.34). There was no evident ^18^F-FDG uptake in the tumor.

When the animals were fasted without being warmed, PET images of the tumors were acquired. However, the images were unstable (2/6) and of a poor quality (Fig. 1C). Fasting only increased the ^18^F-FDG uptake (T/N, 1.153±0.008) of the tumor to a certain degree. When the mice were warmed and fasted, tumor images of a satisfactory quality were acquired (12/12; Fig. 1D–F). The results showed that warming and fasting in combination significantly increased the ^18^F-FDG uptake of the tumor (T/N, 2.0±0.29) and reduced the ^18^F-FDG uptake of the brown fat and myocardium compared with the mice that were only fasted (P<0.05).

### Impact of ^18^F-FDG uptake duration on image quality

In this study, the effect of the ^18^F-FDG acquisition time was analyzed. Intriguingly, the duration of ^18^F-FDG uptake had no impact on the tumor image quality. Following intraperitoneal injection of ^18^F-FDG for 1, 1.5 and 2 h, the PET images of the tumors were displayed. There were no statistical differences in the T/N radios among the three groups (1.904±0.246, 2.114±0.354 and 1.978±0.312, respectively; P>0.05).

## Discussion

When PET scanning was introduced at the end of the 1970s, its metabolic/functional image qualities led to an immediate interest. The available tracers made it possible to study blood flow, regional oxygen consumption, the main metabolic pathways and ligand-receptor interactions in the brain, heart and numerous types of tumor (4). At present, PET scanning is predominantly used in oncology, due to its noninvasive, quantitative and reproducible characteristics. During the diagnosis of a number of different types of cancer, including lung, breast and thyroid carcinomas, ovarian cancer, brain, head and neck and bone tumors and lymphoma, PET has demonstrated significant advantages. PET scanning, with the glucose analog FDG, is also increasingly used to study murine models of human diseases. It exhibits a powerful evaluation modality in experimental research for monitoring the progression and transformation of tumors (5), the biological characterization of tumor tissue (6) and for determining the efficacy of therapeutic agents (7). However, due to the different types of tumors and tumor heterogeneity, there is a diversity among the images produced. In order to understand the imaging characteristics of PET scanning for LSCC xenografts, the present study used ^18^F-FDG as a tracer for tumor imaging. However, the mode of anesthesia, the dietary conditions and ambient temperature have been shown to influence the ^18^F-FDG uptake of the tumor. Therefore, the present study investigated the effects of these factors on tumor imaging, and a handling protocol that optimized the quality of the image of the tumor was developed.

The type of anesthesia used was a critical factor in the process of the study. Chloral hydrate, isoflurane and pentobarbital are the most commonly used anesthetics in rodent animal experiments. In this study, the effects of these three types of anesthetics on the mice were compared. The results showed that 7 ml/kg 5% chloral hydrate (the highest limit for common usage) was lethal for the nude mice, while a dosage of 5 ml/kg was only able to induce anesthesia for 30–40 min. The administration of an additional dosage (2 ml/kg) of chloral hydrate was dangerous for the nude mice. The effects of isoflurane inhalation were induced rapidly (≥10 sec); however, the duration of the effects was too short (≥1 min). When combining isoflurane inhalation with chloral hydrate injection, a synergistic effect was observed in the course of the anesthesia. However, the combination of the drugs was shown to be more dangerous. The administration of 1% pentobarbital (5 ml/kg) was relatively safe for the nude mice. The drug acted for a longer duration (40–50 min), and it was possible to administer an additional dose (2 ml/kg) to the mice according to the experimental requirements.

Due to the limitations in spatial resolution, the volume of the tumor may directly affect the quality of the imaging (8). Wu and Yu (9) observed that a tumor diameter between 0.5 and 0.8 cm was suitable for the conduction of interventional research. When the tumor diameter exceeded 1.0 cm, necrosis occurred in the center of the tumor, which had a detrimental effect on the tumor image. However, in a study on the use of clinical PET scanning for human nasopharyngeal carcinoma xenografts, Yuan *et al*(10) observed that a maximum tumor diameter of between 1 and 1.4 cm was more appropriate for clinical PET imaging. With regard to these studies, we selected tumor diameters between 0.8 and 1.5 cm as the objects of ^18^F-FDG PET study. The results showed that tumor diameters in this range were suitable for PET imaging. There was no significant correlation between tumor size and PET image quality.

The conditions in which the animals are kept have a significant impact on the ^18^F-FDG uptake of the tumor. Various handling conditions, such as fasting and warming, may lead to different tumor image qualities, with the difference of image quality between warming and no warming being approximately two-fold. Fueger *et al*(11), who studied the impact of animal handling on the result of ^18^F-FDG PET imaging using micro-PET, observed that fasting and warming resulted in over a three-fold increase in tumor ^18^F-FDG uptake. This may have been associated with glucose levels. When animals were allowed free access to food, the insulin levels of the mice were increased, which induced the elevation of blood sugar. Glucose may compete with ^18^F-FDG for intracellular uptake and phosphorylation and thereby reduce the ^18^F-FDG uptake of the tumor. Furthermore, the elevated insulin levels may result in an increased ^18^F-FDG uptake by skeletal muscle and the myocardium (12,13), in addition to enhancing the metabolic activity of brown adipose tissue (14). These factors may result in the enhancement of the images of these tissues, but not the tumor. However,, sufficient fasting may increase the ^18^F-FDG uptake of the tumor and reduce the activity of muscle, heart and brown adipose, thereby improving the tumor image quality.

Ambient temperature is also an important factor with regard to ^18^F-FDG uptake. Data have shown that ambient temperature has a pronounced effect on ^18^F-FDG biodistribution in mice; for mice, the optimal ambient temperature lies between 30 and 34ºC (15). At this temperature, body temperature is maintained by heat convection and no additional activity is required. However, when the temperature is decreased, brown adipose tissue and muscle activity are required to generate heat, in order to maintain a constant body temperature. High temperature may increase the heart rate, leading to dehydration of the animal, hypoglycemia and renal injury (16). Therefore 30ºC was adopted as the ambient temperature in this study. The results showed that this temperature was appropriate and resulted in the production of clear tumor images.

The image acquisition time was an additional important factor. The transformation rate from hexokinase (HK) to glucose-6-phosphate (G6P) has been shown to be different among malignant and benign tumors and inflammatory lesions, while the ^18^F-FDG uptake rates have also been shown to differ accordingly (17). According to the Warburg effect, the ^18^F-FDG uptake of a tumor is high; therefore, conducting PET scanning too early or too late may influence the tumor imaging. In clinical practice, patients usually undergo FDG-PET following the intravenous injection of ^18^F-FDG for ~1 h. To distinguish between tumors and inflammation, delayed imaging may be performed in patients with tumors, by injection with ^18^F-FDG for 2 h (18). According to clinical procedure, the present study adopted 1, 1.5 and 2 h of ^18^F-FDG injection as the initiation time for the PET scanning. The results showed that the acquisition time did not have any impact on the quality of the image of the tumor. This was due to the fact that following 1 h of ^18^F-FDG injection, the uptake of the tumor was high and was maintained for ~1 h.

In conclusion, clinical ^18^F-FDG PET scanning may be applied to LSCC xenografts in a nude mouse animal model. The results of the present study showed that a tumor diameter of 0.8–1.5 cm is appropriate for PET scanning; that overnight fasting and warming are critical conditions for tumor imaging and that the animals should be quiet and relaxed, which may be achieved by the administration of 1% pentobarbital. In addition, the results showed that injection of ^18^F-FDG for 1 h is an appropriate injection duration for PET scanning.

## Figures and Tables

**Table I:** Summary of experimental conditions.

Group	n	Fasting	Warming	Anesthetic	Scanning start time (h)
A	3	Yes	No	Chloral hydrate	0
B	3	Yes	No	Isoflurane, Chloral hydrate	0
C	3	No	No	Pentobarbital	1
D	6	Yes	No	Pentobarbital	1
E	4	Yes	Yes	Pentobarbital	1
F	4	Yes	Yes	Pentobarbital	1.5
G	4	Yes	Yes	Pentobarbital	2
